# AMPK, a key regulator of metabolic/energy homeostasis and mitochondrial biogenesis in cancer cells

**DOI:** 10.1038/cddis.2015.404

**Published:** 2016-01-14

**Authors:** B Chaube, M K Bhat

**Affiliations:** 1 Laboratory No. 6, National Centre for Cell Science, Savitribai Phule Pune University Campus, Ganeshkhind, Pune, Maharashtra 411007, India

Normal cellular physiology requires sufficient supply of nutrients for the generation of ATP, building blocks and reducing capacity. In contrast, rapid proliferation of tumor cells demands increased requirement of the fundamental building blocks, which in turn necessitates augmented utilization of nutrients as a carbon source.^1^ However, owing to poor vasculature, availability of carbon and nitrogen sources and molecular oxygen is compromised, which leads to metabolic stress in solid tumors.^2^ Under such conditions, intrinsically altered cancer cell metabolism generates heterogeneity in the concentrations of glucose and other metabolites, as well as oxygen and pH across the tumor.^2^ Therefore, metabolic adaptation to altered and insufficient availability of nutrients and oxygen is critical for overall tumor progression. These conditions persuade a series of changes in the molecular or biochemical pathways, mediating the cellular stress responses under extreme conditions in tumor microenvironment, which are poorly understood.

In a recent study published in *Cell Death and Discovery*, it has been shown that energy homeostasis and adaptation to metabolic stress in cancer cells are primarily achieved by integrated response exerted by activation of an energy sensor, AMPK.^3^ The study provides evidence that the AMPK–p38–PGC1-*α* axis, by increasing mitochondrial biogenesis, supports oxidative metabolism of non-glucose substrates to maintain the cellular ATP pool. This study highlights the fundamental role of AMPK in maintaining energy and metabolic homeostasis, and in promoting cell survival under glucose-limiting condition.

AMPK is a hetero-trimeric protein complex that plays an essential role in the regulation of metabolic and energy homeostasis.^4^ AMPK, by regulating metabolism and critical biological functions, conserves cellular energy and viability during metabolic stress condition.^4^ Though the role of AMPK in regulating energy homeostasis and cell survival in normal tissues is well understood,^4^ its role in cancer remains contradictory.^5^ It has been shown that AMPK, by controlling an array of molecular as well as biochemical pathways, restores the energy and metabolic status, which is crucial for survival of cancer cells.^3^ Importantly, AMPK-mediated cell survival requires inhibition of mTOR. As mTOR mainly regulates anabolic processes, its activation can perturb energy homeostasis under metabolic stress (Figure 1).^3, 6^ Inhibition of mTOR augments cellular ATP level, which positively correlates with cell survival even in the absence of AMPK.^3^ While AMPK and mTOR play antagonistic role in cells, inhibition of mTOR is essential for AMPK-mediated metabolic homeostasis.

Although aerobic glycolysis is the well-recognized metabolic phenotype of cancer cells, oxidative phosphorylation (OXPHOS) is not completely inactivated even in glycolytic tumors.^7^ Clinical FDG–PET data, as well as preclinical *in vitro* and *in vivo* data, clearly suggest that cancer cells are proficient in using alternate carbon sources.^7^ Amino acids such as glutamine, serine, glycine, fatty acids and even glycolytic waste lactate can be used as fuels by tumor cells under certain genetic alterations and during metabolic stress.^8^ Importantly, activation of AMPK is necessary to facilitate oxidative metabolism of non-glucose substrates, specially glutamine and lactate, to maintain cell survival.^3^ This could be an alternative mechanism for restoring cellular ATP level required for cancer cell survival under glucose-limiting conditions (Figure 1).

Enhanced mitochondrial functionality is a prerequisite for generation of more ATP under metabolic stress condition. It has been reported that mitochondrial biogenesis and activities of respiratory enzymes are enhanced in muscle cells in response to chronic energy deprivation.^9^ It is evident from this study that activation of AMPK is decisive for mitochondrial biogenesis to compensate for the glucose-limiting conditions in cancer cells.^3^ Moreover, AMPK-dependent increased mitochondrial OXPHOS capacity facilitates generation of ATP from non-glucose substrates in cancer cells under metabolic stress. Activation of AMPK enhances expression of PGC1-*α* and its target genes in cancer cells under glucose-limiting conditions.^3^ Moreover, inhibition of mTOR by rapamycin increases the expression of PGC1-*α*,^10^ suggesting that mTOR inversely correlates with PGC1-*α* expression, and it might be a negative regulator of mitochondrial biogenesis.^3^ Activation of AMPK with concomitant inhibition of mTOR is decisive in maintaining energy homeostasis and mitochondrial function (Figure 1).

Stress kinase, p38MAPK, by regulating expression of PGC1-*α*, promotes mitochondrial biogenesis in muscles.^11^ However, the role of p38 in metabolic homeostasis in cancer cells is not clearly understood. Recently, it has been reported that p38 activity is required for AMPK-mediated cell survival under metabolic stress, and these two can regulate expression of PGC1-*α*.^3^ Though activation of p38 is necessary for AMPK-induced PGC1-*α* expression, its inhibition, however, does not affect AMPK activation, but it causes reduction in the levels of PGC1-*α* and TFAM (Figure 1).^3^ This suggests that p38 mediates AMPK-induced mitochondrial biogenesis.

Adaptation to various metabolic stress in metabolically heterogeneous tumor microenvironment is indispensable for cancer cell survival.^3, 12, 13^ Evidences indicate that cancer cells respond to changes in the concentrations of glucose and other nutrients by altering mitochondrial metabolism/biogenesis.^3^ Under glucose-limiting condition, mitochondrial activity is elevated, which can be restored to normal level upon re-feeding cells with glucose.^3^ Interestingly, cells lacking functional AMPK are unable to adapt to fluctuating nutrient concentrations, which causes cell death owing to the perturbation in energy homeostasis.^3^ Speeding up the rate of glycolytic reactions for generating more ATP is an adaptive response of cells to maintain energy homeostasis under metabolic stress.^14^ AMPK, by phosphorylating PFK2, enhances glycolysis, which constitutes a homeostatic mechanism for quick restoration of the ATP pool in cancer cells under metabolic stress (Figure 1).^3^

Conclusively, the study by Chaube *et al.* highlights the role of AMPK in controlling cellular bioenergetics and metabolic homeostasis in cancer cells. AMPK is an important proximal signaling step for regulating mitochondrial biogenesis, and ensuring cell survival under metabolic stress via regulating p38/PGC1-*α*. AMPK regulates both glycolysis as well as mitochondrial biogenesis depending upon the nutrient availability (Figure 1).^3^

## Figures and Tables

**Figure 1:**
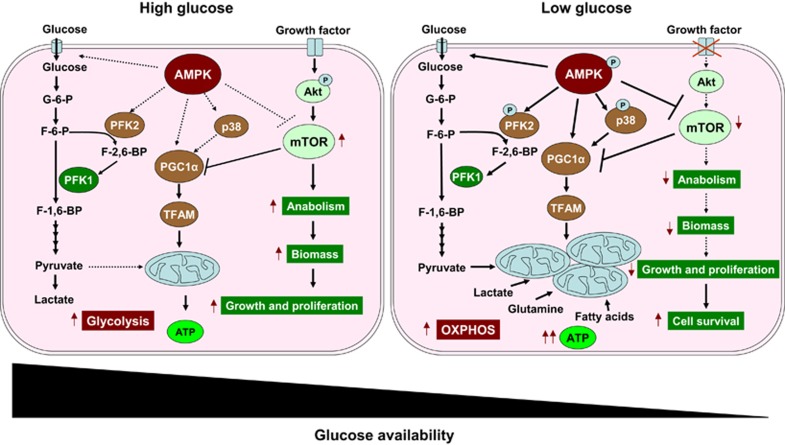
AMPK regulates metabolic and energy homeostasis in cancer cells. Under nutrient-abundant condition, AMPK is suppressed and mTOR is activated, which promotes generation of biomass that leads to cell growth and proliferation. While under glucose-limiting condition, AMPK, by activating p38 and by inhibiting mTOR, regulates expression of PGC1-*α*, which controls mitochondrial biogenesis in cancer cells, thereby allowing oxidative metabolism of non-glucose carbon sources, such as glutamine, lactate and fatty acids, to generate ATP. Simultaneously, AMPK can elevate the rate of glycolysis by activating PFK2 and glucose utilization to maintain metabolic homeostasis. (Thick lines represent activated state and dotted lines represent inactivated state.) F-6-P, fructose 6-phosphate; F-1,6-BP, fructose 1,6-bisphosphate; G6P, glucose 6-phosphate
